# Preparation of PLGA Nanoparticles by Milling Spongelike PLGA Microspheres

**DOI:** 10.3390/pharmaceutics14081540

**Published:** 2022-07-24

**Authors:** Jimin Lee, Hongkee Sah

**Affiliations:** College of Pharmacy, Ewha Womans University, 52 Ewhayeodaegil, Seodaemun-gu, Seoul 03760, Korea; intellimin@ewhain.net

**Keywords:** poly-*d*,*l*-lactide-*co*-glycolide, microspheres, nanoparticles, wet milling

## Abstract

Currently, emulsification-templated nanoencapsulation techniques (e.g., nanoprecipitation) have been most frequently used to prepare poly-*d*,*l*-lactide-*co*-glycolide (PLGA) nanoparticles. This study aimed to explore a new top-down process to produce PLGA nanoparticles. The fundamental strategy was to prepare spongelike PLGA microspheres with a highly porous texture and then crush them into submicron-sized particles via wet milling. Therefore, an ethyl formate-based ammonolysis method was developed to encapsulate progesterone into porous PLGA microspheres. Compared to a conventional solvent evaporation process, the ammonolysis technique helped reduce the tendency of drug crystallization and improved drug encapsulation efficiency accordingly (solvent evaporation, 27.6 ± 4.6%; ammonolysis, 65.1 ± 1.7%). Wet milling was performed on the highly porous microspheres with a D_50_ of 64.8 μm under various milling conditions. The size of the grinding medium was the most crucial factor for our wet milling. Milling using smaller zirconium oxide beads (0.3~1 mm) was simply ineffective. However, when larger beads with diameters of 3 and 5 mm were used, our porous microspheres were ground into submicron-sized particles. The quality of the resultant PLGA nanoparticles was demonstrated by size distribution measurement and field emission scanning electron microscopy. The present top-down process that contrasts with conventional bottom-up approaches might find application in manufacturing drug-loaded PLGA nanoparticles.

## 1. Introduction

PLGA nanoparticles have attracted much interest in nanomedicine [1]. They find applications in the sustained delivery of antigens, drugs, nucleic acids, and proteins. Additionally, functionalized PLGA nanoparticles have been widely used to target tumors or specific organs [2]. The best attribute of PLGA comes from the fact that worldwide, regulatory agencies currently approve it in various dosage formulations for parenteral drug delivery systems [3]. The polymer shows excellent biocompatibility and biodegradability in the human body.

Most drug-containing PLGA nanoparticles are prepared using emulsification-templated nanoencapsulation techniques [4,5]. Representative methods include emulsion solvent evaporation/extraction, nanoprecipitation, salting-out, membrane emulsification, microfluidic technology, and the flow focusing technique. The most used manufacturing process among the above methods is nanoprecipitation [6,7,8,9,10,11]. A kinetically stable nanosuspension appears spontaneously when a PLGA/drug/solvent dispersed phase is emulsified with an aqueous phase under a sink condition. Nanoprecipitation does not need the use of high-energy or high-pressure mixing devices because the formation of PLGA nanoparticles is driven by the spontaneous diffusion of an organic solvent into water. This so-called Ouzo effect is a process that causes the formation of nanoemulsion droplets into solid polymeric nanoparticles. Sometimes, this nanoprecipitation method is named the solvent displacement technique [12,13]. Another important fact is that the mode of mixing a polymeric dispersed phase with an antisolvent influences the quality of PLGA nanoparticles. Therefore, a microfluidic device is sometimes used for mixing [14]. The solvent used in this practice should be water-miscible. It can be removed from a nanosuspension by dialysis, diafiltration, evaporation, extraction, or a combination of these. Among various solvents, acetone is the preferred choice of solvent in the practice of nanoprecipitation.

A high-energy mixing device is required in the emulsion solvent evaporation/extraction manufacturing process using ethyl acetate or methylene chloride [15,16,17,18]. Moreover, the size of polymeric nanoparticles is generally larger than those prepared by nanoprecipitation. Finally, the emulsification-templated nanoencapsulation process is a bottom-up process. PLGA and a hydrophobic drug are dissolved in an organic solvent to prepare nanoemulsion droplets, and then, solid PLGA nanoparticles are manufactured by curing them.

A top-down approach for manufacturing drug nanocrystals involves crushing larger solid drug particles into nano-sized particles. Particle size reduction is usually achieved by media milling, high-pressure homogenization, or microfluidization [19,20]. Nanomilling, referring to reducing the size of particulate matter to a nanoscale, is performed mainly in an aqueous solution, using milling media (beads, balls, or pearls). It is one valuable technology platform for developing nanomedicines [21,22,23,24,25,26,27]. It is tough to make hydrophobic drug particles of 1 μm or less by a dry milling method. In the case of manufacturing nano-sized particles, the grinding medium is put into an aqueous phase together with drug powders. Subsequently, the entire system is subject to milling. Nanocrystal is the most representative name for the underlying technology. Commercial drug products developed using this technology include Rapamune, Emend, Megace ES, Tricor, Invega Sustenna, Ritalin LA, and Zanaflex.

A bottom-up nanofabrication technique is a method in which nanoparticle-forming materials are entirely dissolved in a solvent and then fabricated to nano-scale particles by precipitation, self-assembly, spray drying, or freeze drying [20,28]. Currently, the methods of manufacturing PLGA nanoparticles rely on a bottom-up process, as described earlier. This study aimed to develop a top-down approach for manufacturing PLGA nanoparticles for the first time. Spongelike PLGA microspheres containing a hydrophobic drug were first manufactured following an ammonolysis-based microencapsulation process. The possibility of manufacturing nanoparticles by milling the dried spongelike PLGA microspheres was then explored in this study. Nonhalogenated ethyl formate, belonging to ICH class 3 solvents, was used as a PLGA-dissolving solvent. Progesterone was used as a model drug throughout this study.

## 2. Materials and Methods

### 2.1. Materials

Poly-*d*,*l*-lactide-*co*-glycolide (RG502H; lactide:glycolide molar ratio, 50:50; inherent viscosity of a 0.1% concentration in CHCl_3_, 0.20 dL/g) was purchased from Evonik Corporation (Theodore, AL, USA). This polymer, abbreviated as PLGA in the text, was used as a microsphere-forming material. Poly (vinyl alcohol) (PVA; 88 mol% hydrolyzed; molecular weight, 25,000 g/mol) was obtained from Polysciences, Inc. (Warrington, PA, USA). Progesterone, ethyl formate, acetone, hydroxypropyl methylcellulose (HPMC; viscosity of a 2% aqueous solution, 40~60 cP), and Tween 80 were procured from Merck Korea (Seoul, Korea). A 28% ammonia solution was obtained from Junsei Chemical Co., Ltd. (Tokyo, Japan). Methanol and tetrahydrofuran were supplied from Honeywell Korea (Seoul, Korea).

### 2.2. Preparation of PLGA Nanoparticles by Nanoprecipitation

PLGA (0.3 g) and progesterone (30 mg) were dissolved in 6 mL of acetone. This dispersed phase was injected into 30 mL of a 0.5% PVA solution being stirred at 450 rpm by a digital magnetic stirrer. The stirring continued for 4 h at room temperature (RT). After that, the nanosuspension was centrifuged at 25,000× rpm for 1 h at 4 °C (model Optima LE-80K; Beckman Coulter Inc., Brea, CA, USA). Pellets were dried in a vacuum dryer overnight.

### 2.3. Preparation of Nonporous PLGA Microspheres by a Solvent Evaporation Process

Nonporous microspheres containing progesterone were prepared by an emulsion solvent evaporation process using ethyl formate. PLGA (0.3 g) and progesterone (30 or 60 mg) were dissolved in ethyl formate (6 mL). This dispersed phase was poured into 30 mL of a 0.5% aqueous PVA solution being stirred at 450 rpm with a digital magnetic stirrer (model 400 series; VWR Scientific, Radnor, PA, USA). The nascent microsphere suspension was mixed with 100 mL of a 0.1% PVA solution, which was further stirred for 2 h at RT. The resultant microsphere suspension was passed through two sieves with different pore sizes (425 and 25 μm). This wet sieving was performed to separate microspheres from unentrapped progesterone crystals. The microspheres trapped between the two sieves were thoroughly washed with water and collected through filtration. The microspheres were vacuum dried overnight at ambient temperature. For a given microsphere manufacturing formulation, microspheres were prepared in triplicate.

### 2.4. Preparation of Porous PLGA Microspheres by an Ammonolysis Process

Porous PLGA microspheres with a spongelike morphology were prepared by a modified ammonolysis-based single emulsion technique reported elsewhere [29]. A dispersed phase consisting of 4 mL of ethyl formate, 0.3 g of PLGA, and 30~60 mg of progesterone was emulsified in 30 mL of a 0.5% PVA solution using a digital hotplate stirrer. After 3 min stirring at RT, a 28% ammonia solution (10 mL) was added to the oil-in-water (o/w) emulsion. Ammonolysis of ethyl formate in the dispersed phase led to water-soluble ethanol and formamide formation. As they served as anti-solvents toward PLGA, oil droplets were quickly hardened into solid microspheres. Their leaching into the aqueous phase resulted in numerous macro-pores across the microsphere matrices. After 5 min stirring, the microsphere suspension was poured into 100 mL of a 0.1% PVA solution being stirred at 450 rpm. After 15 min, the microsphere suspension was passed through two sieves with different pore sizes (425 and 25 μm). The microspheres trapped between the two sieves were redispersed in 100 mL of the 0.1% PVA solution, stirred at 350 rpm for 90 min, and collected by filtration. Microspheres were vacuum dried overnight. Three batches were prepared for a given formulation.

### 2.5. Observation of Particulate Suspensions by Optical Microscopy

The state of the embryonic microsphere suspension during microencapsulation was monitored by a light microscope (LM; model Axioscope A1, Carl Zeiss Microscopy GmbH, Jena, Germany). Critical attributes of interest were microsphere morphology (e.g., size) and the tendency of progesterone crystallization occurring in an aqueous phase. The light microscope was also used to observe how the size of the microspheres changed during wet milling.

### 2.6. Measurement of Microsphere Size Distribution

The size distribution of a porous, spongelike microsphere suspension was measured by the Mastersizer 3000E (Malvern Instruments Ltd., Worcestershire, UK). The microsphere suspension appearing in the ammonolysis-based process was subjected to wet sieving and diluted with distilled water to a final volume of 540 mL. The final microsphere suspension was placed inside the particle size analyzer.

### 2.7. Determination of Drug Encapsulation Efficiency

Dried microspheres (20~30 mg) were dissolved in 4 mL of tetrahydrofuran. An aliquot of this solution (200 μL) was diluted 10 times with a mixture of methanol and water (8:2, by *v/v*). This solution was filtered through a 0.45 μm nylon filter, and the filtrate was used as the HPLC sample. A Luna 5 μm C18 (250 × 4.6 mm, 100Å) was used as the analytical column, whereas a methanol-water mixture was the mobile phase. Its flow rate was set at 0.8 mL/min, and progesterone eluting from the column was detected at 254 nm. The progesterone encapsulation efficiency (EE%) was determined using the equation below:

Progesterone EE% = (actual drug loading ÷ theoretical drug loading) × 100

where theoretical drug loading = (amount of progesterone used/total amounts of PLGA and progesterone used); actual drug loading = (measured content of progesterone/amount of microsphere sample used for analysis). 

### 2.8. Scanning Electron Microscopy (SEM)

The morphology of PLGA microspheres was observed using a scanning electron microscope (model JSM-5200; Jeol Inc., Tokyo, Japan). Microspheres were sprinkled over double-sided adhesive tape attached to a cylinder specimen stub. This specimen stub was sputter-coated with platinum under vacuum in an argon atmosphere (model SC7620 sputter coater; VG Microtech, West Sussex, UK). The inside of the porous microspheres was observed through the following method. Scotch tape was applied to the above double-sided adhesive tape on which microsphere samples were dispersed. The tape composite was then lightly pressed, and the Scotch tape was detached. At this time, a part of the microsphere surface was peeled off. This procedure led to the exposure of the inside of the microspheres. This specimen stub was sputter-coated as described earlier.

### 2.9. Field Emission-Scanning Electron Microscopy (FE-SEM)

A PLGA nanosuspension was centrifuged at 25,000× rpm at 4 °C for 1 h (model Optima LE-80K; Beckman Coulter, Seoul, Korea). The morphology of the PLGA nanoparticles was observed with an FE-SEM (model JEOL-7800F; Jeol Ltd., Tokyo, Japan). The particulate samples were dispersed on an iron mount to which carbon tape was attached. The specimen mount was coated with platinum in a vacuum using a sputter coater (model Cressington 208HR; Cressington Scientific Instruments Ltd., Watford, UK).

### 2.10. Wet Milling

The planetary mill Pulverisette 7 (Fritsch GmbH, Idar-Oberstein, Germany) was used to grind porous PLGA microspheres into smaller particles. The grinding bowl size was 20 mL. Dried microspheres (30 mg) were suspended in 8 mL of an aqueous solution containing 0.5% HPMC and 0.1% Tween 80. The microsphere suspension was put into the grinding bowl containing 80 zirconium oxide beads with a diameter of 5 mm. After milling at 500 rpm for 10 min, the milling chamber was cooled at 5 °C for 20 min. This cycle was repeated 60 times. As a consequence, the actual milling time totaled up to 10 h. After the grinding beads were replaced with 3 mm zirconium beads (total of 30 g), the system was subject to milling at 600 rpm for 10 min and cooling at 5 °C for 20 min. This cycle was repeated 30 times, so that the actual milling time reached 5 h. At the end of milling, a sieve with a pore size of 250 μm was used to remove the grinding beads. The resultant particulate suspension was filtered through a nylon filter with a pore size of 5 μm.

### 2.11. Viscosity Measurement

Solvent viscosity is a critical factor for the measurement of nanoparticle size. Lacking accurate viscosity values leads to erroneous diffusion coefficient calculation during dynamic light scattering measurements. The viscosity of our solvent (i.e., an aqueous solution containing 0.5% HPMC and 0.1% Tween 80) was measured using a rotational Brookfield DVE digital viscometer (AMETEK Brookfield; Middleboro, MA, USA). The test method specified in ASTM-D2196 was used to determine the apparent viscosity (spindle 00, a rotational speed of 100 rpm, torque 56.2%).

### 2.12. Size Measurement of PLGA Nanoparticles

A dynamic light scattering particle analyzer (model Zetasizer Nano ZS90; Malvern Instruments Ltd., Worcestershire, UK) was used to measure the size distribution of PLGA nanoparticles prepared by wet milling. An aliquot (100 μL) of the PLGA nanosuspension was placed in a ZEN0118 cuvette, and the light was scattered at 90°.

## 3. Results and Discussion

PLGA nanoparticles are frequently manufactured by emulsification-solvent evaporation/extraction and nanoprecipitation techniques. It was reported elsewhere that the quality attributes of PLGA nanoparticles were tuned by adjusting process parameters such as PLGA concentration, solvent type, the dispersed phase-continuous phase ratio, temperature, the injection speed of a dispersed phase to a continuous phase, and mixing condition [6,7]. Huang and Zhang hypothesized that these parameters affected D_pw_ (the diffusion coefficient of a dispersed solvent in water), thereby controlling the size of PLGA nanoparticles [7]. For example, acetonitrile with a higher D_pw_ produced smaller PLGA nanoparticles with narrower size distribution than tetrahydrofuran with a lower D_pw_.

**Figure 1:**
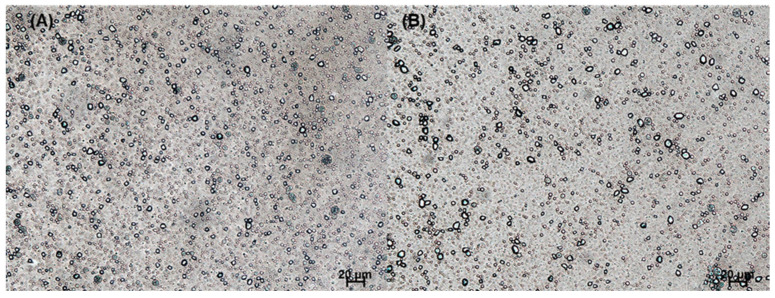
Light microscopy (LM) photographs showing the appearance of non-spherical progesterone crystals formed in the nanoprecipitation process. After injecting a polymeric dispersed phase into an aqueous phase, the status of the resultant mixture was observed at (**A**) 5 min and (**B**) 4 h. The bar size is 20 μm.

**Figure 2:**
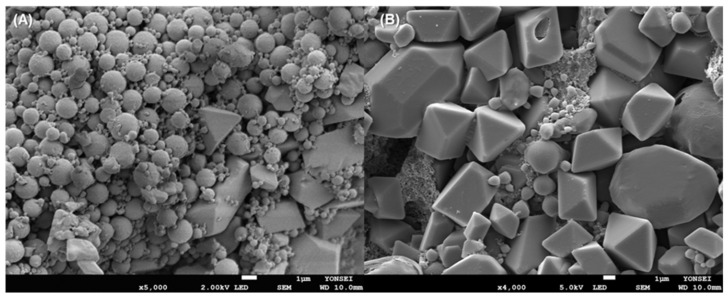
SEM images of particles collected by ultracentrifugation. (**A**) PLGA particles are contaminated with non-spherical progesterone crystals of various sizes. (**B**) is a close-up micrograph showing progesterone crystals. The bar size is 1 μm. The initial progesterone payload used for nanoencapsulation was 30 mg.

If drug crystals and PLGA nanoparticles exist in a nanosuspension, various quality problems will occur. However, it has been largely overlooked that some hydrophobic drugs tend to precipitate as crystals in the aqueous phase during nanoprecipitation. In our study, a large portion of progesterone was not encapsulated inside PLGA particles. Figure 1 is a light microscopic image of the nanosuspension fabricated through our nanoprecipitation process. Non-spherical progesterone crystals dispersed in the aqueous phase were observed immediately after mixing the dispersed phase with the aqueous phase and at the end of the nanoprecipitation process. The SEM images in Figure 2 demonstrate the presence of drug crystals of different sizes in PLGA particles.

**Figure 3:**
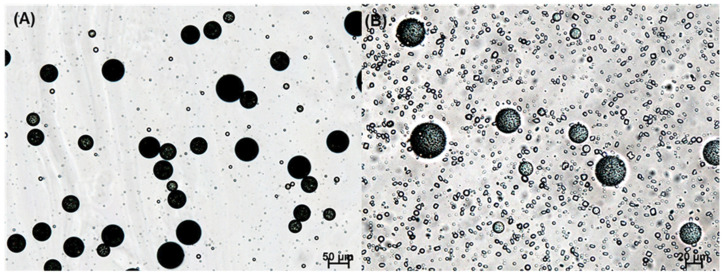
LM photographs of the emulsions that were obtained by emulsifying a dispersed phase (PLGA 0.3 g, progesterone 30 mg, ethyl formate 6 mL) in an aqueous phase. The emulsion was stirred for (**A**) 10 min and (**B**) 2 h before LM observation. The bar sizes in (**A**,**B**) are 50 and 20 μm, respectively.

In this study, our initial attempt was to prepare PLGA microspheres by an ethyl formate-based solvent evaporation process and convert them into PLGA nanoparticles by wet milling. However, during the manufacturing process, a large portion of progesterone was not encapsulated in the microspheres; they crystallized in the aqueous phase (Figure 3). An excellent emulsion was observed after an oil phase was emulsified in an aqueous phase. After 2 h, however, numerous progesterone crystals were observed everywhere. This phenomenon is in line with the earlier reports that hydrophobic drugs precipitated as crystals in the aqueous phase when an emulsion-based solvent evaporation/extraction process was carried out to produce PLGA microspheres [30,31,32]. Other authors also reported that the shape of drug crystals during the emulsion solvent evaporation process was affected by the type of emulsifier [33]. In our view, the most decisive factors were the rate at which the emulsion droplets hardened into solid microspheres and the amount of organic solvent present in the aqueous phase. Due to prominent drug crystallization, its encapsulation efficiency (EE) was expected to be very low. Before determining drug EE, a separation process should be performed to collect only PLGA microspheres. The wet sieving procedure described in the experimental section was found to be successful in removing drug crystals from the microsphere suspension.

**Figure 4:**
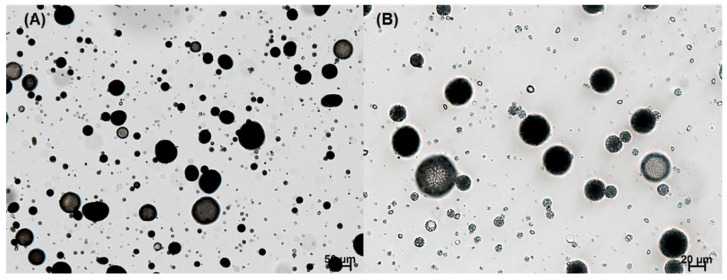
LM photographs of a microsphere suspension that was obtained by the ammonolysis-based microencapsulation process. The initial progesterone payload was 30 mg. Just before wet sieving, the microsphere suspension was sampled for LM observation. The bar sizes in (**A**,**B**) are 50 and 20 μm, respectively.

Spongelike PLGA microspheres were prepared following an ammonolysis-based microencapsulation process to reduce the tendency of drug crystallization. It was also expected that the porous microspheres could be milled more easily than nonporous ones. In a previous study, an ammonolysis microencapsulation technique using isopropyl formate was reported to manufacture spongelike PLGA microspheres with high porosity [29]. The present study developed an ethyl formate-based ammonolysis process to encapsulate progesterone into PLGA microspheres. Isopropyl formate had to be substituted with ethyl formate because the former could not solubilize the PLGA used in this study. One advantage of our ammonolysis microencapsulation process is that emulsion droplets are transformed into highly porous solid microspheres in minutes. Therefore, it was expected to reduce the crystallization phenomenon of progesterone by inhibiting its diffusion into the aqueous phase during the manufacturing process. Figure 4 shows the microsphere suspension observed during our ammonolysis-based microencapsulation process. Our microencapsulation process could significantly reduce drug crystallization, when compared to the ethyl formate-based solvent evaporation process (Figure 3).

**Figure 5:**
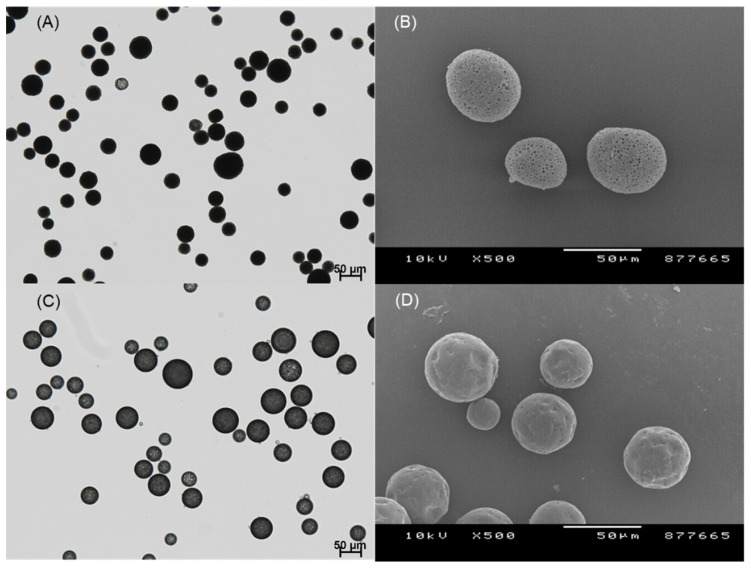
LM photographs of the microsphere suspensions that were prepared by (**A**) ammonolysis and (**C**) solvent evaporation. (**B**) SEM image of the dried microspheres prepared by ammonolysis, and (**D**) produced by solvent evaporation. The bar size in all micrographs is 50 μm.

**Figure 6:**
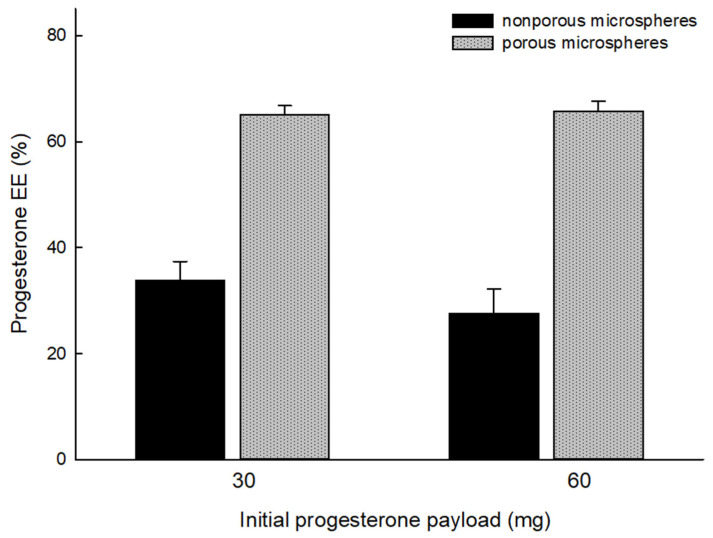
Effect of microencapsulation methods (ammonolysis vs. solvent evaporation) on progesterone EE%. The initial progesterone payload used for microencapsulation varied from 30 to 60 mg, while the PLGA amount was set at 0.3 g.

When the process of microsphere hardening was complete, wet sieving was performed to remove unentrapped drug crystals from the microsphere suspension. It can be seen in Figure 5 that the result of wet sieving was eminently successful. Afterward, drug EE% was determined (Figure 6). The lowest drug EE was 27.6 ± 4.6% when microspheres were prepared through solvent evaporation. In contrast, when spongelike PLGA microspheres were prepared, a drug EE of at least 65.1 ± 1.7% was achieved. These results prove that the ammonolysis-based process reduces the tendency of drug crystallization and improves drug EE accordingly.

**Figure 7:**
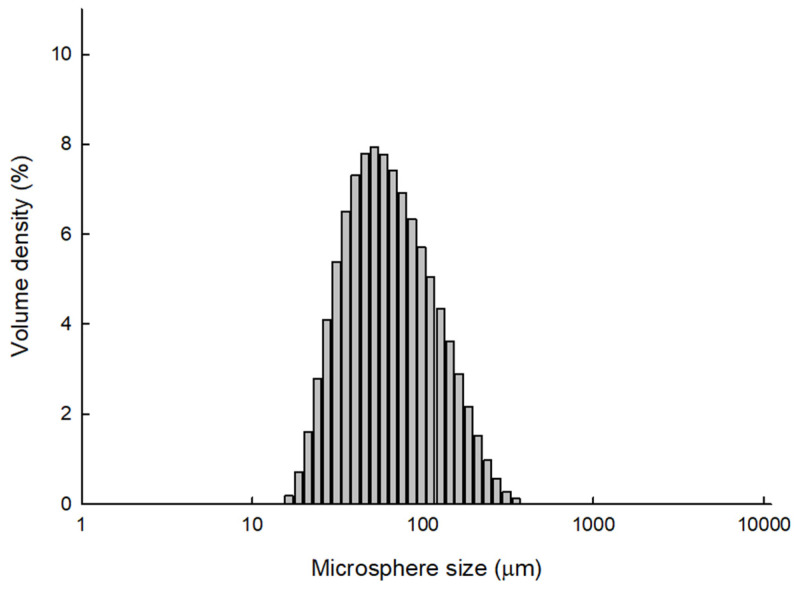
The size distribution of PLGA microspheres that were produced by the ammonolysis-based microencapsulation process. PLGA (0.3 g) and progesterone (30 mg) were used for the microencapsulation process.

**Figure 8:**
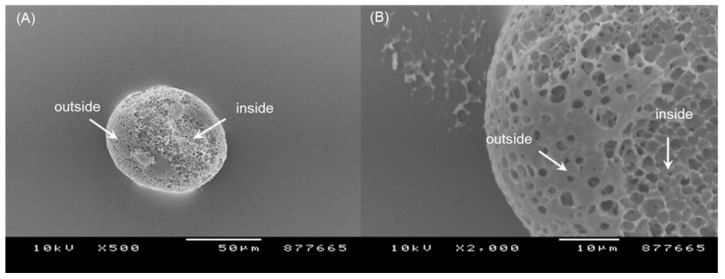
SEM images of the surface and internal morphology of PLGA microspheres prepared by ammonolysis. The initial progesterone payload was 30 mg. The bar sizes in (**A**,**B**) micrographs are 50 and 10 μm, respectively. A tape was used to peel off a portion of the microsphere surface.

Figure 7 shows the size distribution of PLGA microspheres prepared by the ammonolysis process. The values of D_10_ and D_90_ were 31.6 and 157 μm, respectively. D_50_ was 64.8 μm. In addition, no particles with a size of 10 μm or less were measured. Again, these data confirm that progesterone microcrystals were effectively removed through wet sieving (Figure 5). The morphology of the PLGA microspheres prepared using ammonolysis is illustrated in Figure 8. There were countless pores on the microsphere surface. Peeling off a part of the microsphere surface led to the revelation of their inside, which resembled a sponge having a porous texture. Ubiquitous pores were interconnected. The spongelike microspheres with this level of extreme porosity would be more readily crushed than nonporous microspheres with compact matrices.

**Figure 9:**
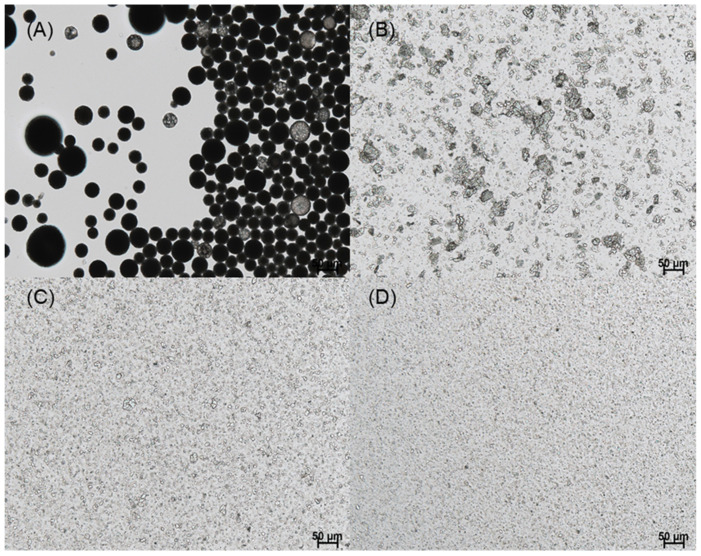
LM micrographs show dynamic changes in the size of spongelike PLGA microspheres as a function of milling time. Beads of 5 mm diameter were used as a grinding medium for (**A**) 0, (**B**) 1, (**C**) 5, and (**D**) 10 h. The bar size is 50 μm.

**Figure 10:**
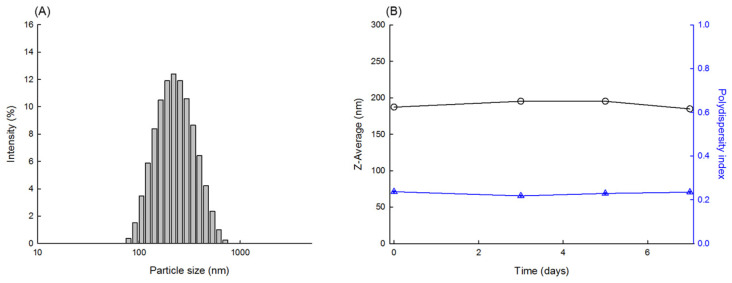
(**A**) The size distribution of PLGA nanoparticles that were prepared by milling spongelike PLGA microspheres. (**B**) Measurements of the Z-average size and PDI of the PLGA nanosuspension while stored at room temperature for a week.

**Figure 11:**
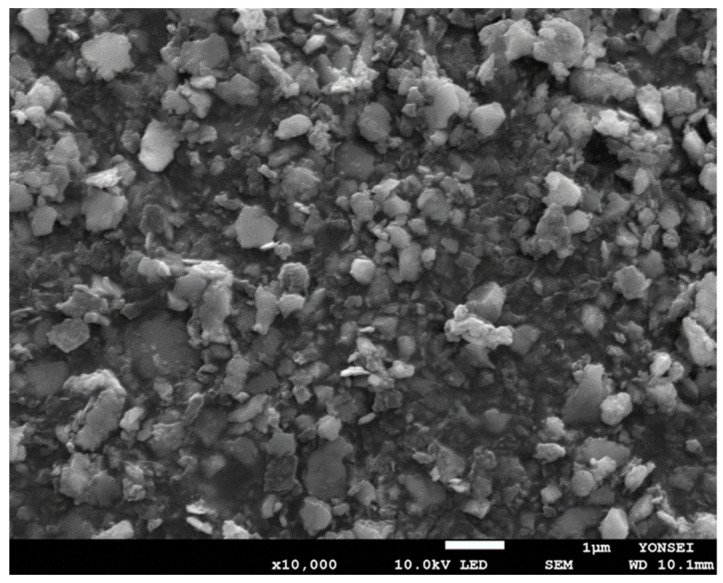
An FE-SEM image of PLGA nanoparticles that were produced by milling pre-formed spongelike PLGA microspheres. The bar size is 1 μm.

Our next attempt was to manufacture PLGA nanoparticles by milling the spongelike PLGA microspheres. HPMC and Tween 80 are often used as stabilizers during milling [21,34]. Based on this point, an aqueous phase containing 0.5% HPMC and 0.1% Tween 80 was selected as the liquid phase in which microspheres and zirconium oxide beads were dispersed. Its viscosity was determined to be 3.37 cP. Milling using smaller zirconium oxide beads ranging from 0.3 to 1 mm was performed on spongelike PLGA microspheres. They were ineffective: the microspheres were not ground into submicron-sized particles. Therefore, larger beads with 3 and 5 mm diameters were chosen as grinding media. Figure 9 shows the dynamic morphological changes in the PLGA microsphere as a function of milling time. The microspheres were crushed into finer particles as milling proceeded. When the milling process was over, the Z-average size of the resultant PLGA nanoparticles was measured to be 187 nm (Figure 10). Its polydispersity index was 0.24, indicating that the size distribution of the nanoparticles was reasonably homogeneous. When stored at RT for a week, there was no considerable change in the size distribution data. The status of the PLGA nanoparticles was also observed through FE-SEM (Figure 11). The nanoparticle size observed with FE-SEM matched with the above Z-average size. The target size of particles is frequently controlled by bead size, bead mass in the milling chamber, and rotor speed. In this study, spongelike PLGA microspheres were effectively ground only when using larger beads. Since significant heat was generated during milling, providing enough cooling to the milling chamber between milling cycles is an imperative that cannot be ignored.

## 4. Conclusions

So far, emulsification-templated nanoencapsulation techniques such as nanoprecipitation have been most commonly used to prepare PLGA nanoparticles. One of the disadvantages of such manufacturing processes is that a hydrophobic drug is not encapsulated inside the nanoparticles and precipitates as crystals of various sizes. The technique platform to produce PLGA nanoparticles reported in this study contrasts with such bottom-up processes. The thrust of our strategy was to prepare spongelike PLGA microspheres first and mill them to make PLGA nanoparticles. An ammonolysis microencapsulation technique using ethyl formate as a dispersed solvent was successfully developed to fabricate progesterone-containing PLGA microspheres with extreme porosity. Compared to a typical solvent evaporation microencapsulation method, the new preparative process increased the drug encapsulation efficiency more than twice. Under our experimental conditions, spongelike PLGA microspheres with a D_50_ of 64.8 μm were milled into PLGA nanoparticles with a Z-average size of 187 nm. This top-down approach was attempted for the first time, and its preliminary feasibility was proven in this study. It would be meaningful to address chemical/solid-state characterization (e.g., FTIR spectroscopy, X-ray diffractometry) and in vitro performance (e.g., drug release, storage stability) of the PLGA nanoparticles in follow-up studies.

## Data Availability

The data presented in this study are available on request. The corresponding author is Sah, H.
